# Spirituality of Science: Implications for Meaning, Well-Being, and Learning

**DOI:** 10.1177/01461672231191356

**Published:** 2023-08-25

**Authors:** Jesse L. Preston, Thomas J. Coleman, Faith Shin

**Affiliations:** 1 University of Warwick, Coventry, UK; 2 Coventry University, UK; 3 University of Illinois at Urbana-Champaign, USA

**Keywords:** awe, science attitudes, science learning, well-being, meaning, spirituality

## Abstract

Scientists often refer to spiritual experiences with science. This research addresses this unique component of science attitudes—*spirituality of science*: feelings of meaning, awe, and connection derived through scientific ideas. Three studies (*N* = 1,197) examined individual differences in Spirituality of Science (SoS) and its benefits for well-being, meaning, and learning. Spirituality of Science was related to belief in science, but unlike other science attitudes, spirituality of science was also associated with trait awe and general spirituality (Study 1). spirituality of science also predicted meaning in life and emotional well-being in a group of atheists and agnostics, showing that scientific sources of spirituality can provide similar psychological benefits as religious spirituality (Study 2). Finally, Spirituality of Science predicted stronger engagement and recall of scientific information (Study 3). Results provide support for an experience of spirituality related to science, with benefits for meaning, well-being, and learning.

Science is the empirical method of knowing based on systematic observation and theorization. But science is more than cold calculation; it reveals the nature of the world and ourselves, the interconnection between living things, and yields awe-inspiring discoveries and theories that create meaning, feelings of connection, and wonder. We argue here that there is something deeper to the scientific experience—beyond mere cognitive understanding and intellectual agreement—that may be best described as a spiritual experience. As Carl Sagan (1996/2011) wrote,

When we recognize our place in an immensity of light years and in the passage of ages, when we grasp the intricacy, beauty and subtlety of life, then that soaring feeling, that sense of elation and humility combined, is surely spiritual.

This project addresses that important but unstudied component of science attitudes, *spirituality of science*: the experience of meaning, connection, and awe derived from scientific ideas, theories, and the scientific process. Spirituality of science is more than just liking for science, or belief in science as a way of knowing. Rather, spirituality of science reflects the deeply positive transcendent experiences that emerge from interactions with science that include feelings of connection, meaning, and awe. Not all people feel spirituality through science, but those who do may reap some important benefits. This includes direct benefits on engagement in science, and better science learning and performance. And more broadly, the spirituality of science may provide some important advantages that parallel those of religious spirituality, including benefits for general well-being and overall feelings of meaning in life.

## Spirituality and Science

How can science be a source of spirituality? It is sometimes unclear what is meant by spirituality and how it is distinct from religious belief. But religion generally refers to the external factors of belief such as group affiliation and practices, whereas spirituality generally refers to the more internal and personal aspects of belief (Pargament, 1999; Zinnbauer et al., 1997). Definitions of spirituality generally emphasize the role of transcendent emotion, connection, and search for meaning (Emmons, 1999; Piedmont, 1999). In other words, spirituality relates to the experience itself (Saucier & Skrzypińska, 2006; Wixwat & Saucier, 2021).

First, spirituality is characterized by experiences of transcendent emotion (Fredrickson, 2002; Van Cappellen et al., 2016), such as feelings of profoundness and beauty (A. B. Cohen et al., 2010). Awe, an emotion defined by perceptions of wonder and vastness (Keltner & Haidt, 2003; Shiota et al., 2007), is of particular relevance to spirituality. Both religious and nonreligious people feel awe and “small self” in response to spiritual experiences, but nonreligious report spiritual experiences from secular sources, for example, experiences in nature (Preston & Shin, 2017). Experimental manipulations of awe have been shown to increase spiritual activities (Van Cappellen & Saroglou, 2012) and also enhance more abstract concepts of God (Johnson et al., 2019). Spiritual experiences are also characterized by feelings of connection and meaningfulness (De Klerk, 2005; Steger, 2013). The meaning in life is an important human motivation (Heine et al., 2006), and where meaning in life is found it is typically through three elements: having purpose, having significance, and sense-making (Heintzelman & King, 2014; Martela & Steger, 2016). The meaningfulness associated with spirituality can contribute to numerous other positive life outcomes, such as workplace satisfaction (De Klerk, 2005) and addiction recovery (Pardini et al., 2000), as well as numerous studies demonstrating benefits for overall well-being (A. B. Cohen, 2002; Holder et al., 2010) and physical health (Thoresen, 1999; Weaver et al., 2006).

These same characteristics of spirituality can be also derived from experiences with science (Preston, 2011). Science evokes strong feelings of awe (Gottlieb et al., 2015; Valdesolo, Park, & Gottleib, 2016; Valdesolo, Shtulman, & Baron, 2016) via the majesty of grand scientific theories and their implications for understanding the nature of ourselves and the universe. For example, predisposition toward awe is associated with improved scientific thinking (Gottlieb et al., 2015), and manipulations that activate feelings of awe also promote an interest in science learning (McPhetres, 2019). Science likewise can provide a powerful sense of meaning, an important aspect of spiritual experiences, through strong explanatory coherence and structure (Preston & Epley, 2005). That is, scientific theories help one to make sense of one’s life and the universe, and in doing so creates an alternative source to the meaning provided by religion (Preston & Epley, 2009; Rutjens et al., 2013). Among nonreligious people, for instance, science may provide important existential meaning that can buffer against the fear of death in the same way that religious meaning does (Farias et al., 2013).

## Benefits of Spirituality of Science

If spirituality can be experienced through science, this carries potential positive implications. Spirituality of science may be beneficial in promoting engagement and learning in science, and in facilitating well-being and meaning. Feelings of wonder and awe through science have been found to promote interest in learning in science (McPhetres, 2019). Likewise, meaningful engagement and spiritual experiences in science could improve learning and retention, and could also predict better long-term educational outcomes and success in science. spirituality of science might also hold implications for overall happiness and well-being. It has been observed that religious people are generally happier and fare better on life satisfaction measures than nonbelievers (Diener et al., 2011; Koenig & Larson, 2001; Ritter et al., 2014), arguably because religion provides a source of meaning (Steger & Frazier, 2005) which helps buffer stress (Inzlicht et al., 2011) and feelings of helplessness in the face of uncertain events (Valdesolo & Graham, 2014). Such findings could imply that nonreligious people are at a relative disadvantage compared with their religious counterparts and may suffer from lower emotional and psychological functioning from a lack of religious meaning. But other research suggests the relationship between religion and mental health is curvilinear, with high positive well-being indicated for both strongly religious and nonreligious people (Galen & Kloet, 2011). If science serves as a source of spirituality, one important implication is it may provide similar benefits for psychological well-being, even for nonreligious people.

## The Present Research

The present work examines the spirituality of science, defined as the experience of meaning, transcendence, and connection derived from scientific ideas, theories, and scientific process. The potential spiritual component of science has been overlooked in psychological studies of science attitudes, where the literature has focused more on topics such as scientific understanding (e.g., Lombrozo et al., 2008; Shtulman & Walker, 2020), and trust in science (Fiske & Dupree, 2014; Rutjens et al., 2018). But examining the spiritual functions of science to include emotional and meaningful components also deserves empirical attention and may have important implications for science learning and well-being.

We developed a short Spirituality of Science (SoS) to measure individual differences in spiritual experiences with science, informed by the psychological literature on spirituality (Hill et al., 2000; Piedmont, 1999). Study 1 establishes the reliability and validity of this measure by comparing it to related science attitudes and experiences with awe and general spirituality. Subsequent studies use the SoS scale to examine the benefits of spirituality of science as a source of meaning and well-being in nonreligious individuals (Study 2), and its implications for science engagement and performance (Study 3).

## Study 1: What is Spirituality of Science?

The goal of Study 1 was to establish spirituality of science as a construct and introduce the Spirituality of Science (SoS) scale. Individual differences on the spirituality of science scale were compared with measures of *Interest in Science* (Johnson et al., 2019) and *Belief in Science* (Farias et al., 2013), to demonstrate convergent validity with other science attitudes. We also establish divergent validity by comparing spirituality of science with these science attitudes on other measures relevant to spirituality: awe, and spiritual transcendence. spirituality of science is expected to be positively related to the *Spiritual Transcendence Scale* (Piedmont, 1999), and *Dispositional Awe* scale (Shiota et al., 2007), as a central affective component of general spirituality, while other science measures have no predicted relationship with these variables. Measures of *Intellectual Humility* (IH; Davis et al., 2015), and *Need for Cognitive Closure* (Webster & Kruglanski, 1994) were also included to assess a general open versus closed attitude toward new ideas. Intellectual openness should be positively related to science attitudes, including SoS, as the scientific method relies on adaptation to new information, an important scientific value.

### Method

#### Note on All Studies

Data, coding, and analyses for all studies (including pilot and filedrawer studies) can be found at: https://osf.io/jxqzd/?view_only=d86446fbfb4c48048958f0910445c7e3. For online studies using paid platforms, we set a value *N*= 500 participants to maximize power (with 90% power to detect a small correlation *r* = .15). In Study 3 using an undergraduate subject pool, we aimed to collect as many participants as possible, with a minimum of *N* = 165 (90% power to detect a medium correlation *r* = .25).

#### Participants

In all, 500 participants (287 men, 212 women, one nonreporting, *M*_age_ = 36.8 years) were recruited on Amazon MTurk in exchange for a small payment.

#### Measures

Participants were recruited to participate in a study on attitudes. Unless otherwise stated, all scale items were on 7-point Likert-type scales, with endpoints: 1 = *strongly disagree*; 7 = *strongly agree*. Participants completed eight scales, in the order described below, then completed demographic information.

*General Religiosity* (α = .97) is a seven-item scale to measure the strength of religiosity (e.g., “My religious beliefs are very important to me”).

*Spiritual Transcendence* (α = .92) is a twelve-item scale to assess general spiritual experiences and attitudes and emphasizes feelings of transcendence (e.g., “I believe that on some level my life is intimately tied to all of humankind”), adapted from Piedmont (1999) and edited for brevity and clarity.

*Interest in Science* (α = .79) is a five-item scale to measure general interest in scientific topics (e.g., “It is important to me to spend time thinking about scientific topics”), adapted from Johnson et al. (2019). See all items in Table 2.

*Belief in Science* (BIS; α = .90) is a five-item measure of the degree of trust an individual places in science. This scale was based on the scale from Farias and colleagues (2013). But, where some items in the original scale pitted science and religion against each other, for our purposes, we instead focus exclusively on endorsement of science as a way of knowing (e.g., “We should only rationally believe what is scientifically provable”). See adapted scale in Table 2.

SoS (α = .93) is a ten-item measure of transcendent emotion, meaning, and connection through science (e.g., “Science reveals the beauty of the world we live in”), developed by the authors. Development of the scale was informed by the psychological literature on spirituality (Hill et al., 2000; Pargament, 1999; Zinnbauer et al., 1997), and drew on themes from the Spiritual Transcendence scale (STS; Piedmont, 1999 which measures general spirituality through three central themes of: (a) transcendent emotion from spiritual activity, (b) belief in the unity and purpose of life, and (c) sense of connection and responsibility to others. This informed our own conceptualization of SoS, and the spirituality of science scale similarly assessed the spiritual relationship to science along three themes of emotional elevation (e.g., “*thinking about science brings me deep joy*”), meaning (e.g., “*there is an order to science that transcends human thinking*”), and connection (e.g., “*all things are connected through science*”). Some items in the spirituality of science scale were directly adapted from the Piedmont (1999) scale, by modifying wording to apply to science (e.g., “*I have had at least one peak experience*” on the STS was modified for the spirituality of science to become “*I have had a peak experience while engaged in science*”). Additional items in the scale were written for the scale to support the themes of transcendence, meaning, and connection. See all items in Table 1. Pilot-testing revealed the final 10-item scale to have good internal reliability (α = .83), see archived data.

**Table 1:** Scale Statistics and Principal Component Analysis for Spirituality of Science, Study 1.

Item	*Mean* (*SD*)	Inter-item correlation	Factor loading	Extraction
1. Science makes me step outside myself to a larger sense of fulfillment	4.01 (*1.73*)	.78	.832	.692
2. I have had a “peak” experience while engaged in science.	3.07 (*1.78*)	.67	.735	.540
3. Thinking about science brings me deep joy.	3.91 (*1.81*)	.81	.856	.733
4. Science helps to show greater meaning in life.	4.20 (*1.83*)	.78	.828	.685
5. There is an order to science that transcends human thinking	4.03 (*1.81*)	.71	.767	.588
6. I have lost track of time while engaged in science	3.38 (*1.94*)	.71	.774	.599
7. Science makes me feel deeply connected to everything.	3.79 (*1.92*)	.85	.884	.781
8. Science reveals the beauty of the world we live in.	4.81 (*1.78*)	.68	.747	.558
9. All things are connected through science	4.49 (*1.81*)	.72	.778	.605
10. Science is a source of spirituality	3.20 (*1.93*)	.58	.651	.423

*Note*: SD = standard deviation.

*Need for Cognitive Closure* (NCC; α = .80) is a seven-item scale to measure preference for certainty in intellectual situations, adapted from Webster and Kruglanski (1994; e.g., “I dislike questions which could be answered in many different ways”).

*IH* (α = .88) is a six-item measure of openness to new ideas and understanding limits of one’s own understanding, for example, “I reconsider (rethink) my opinions when presented with new evidence” (adapted from Davis et al., 2015).

*Awe* (α = .86) is a six-item measure of individual differences in daily experiences of awe, e.g., “I feel awe” (Shiota et al., 2007).

### Results

#### SoS Scale Statistics

The 10-item spirituality of science scale showed strong internal reliability (α = .93), and the mean was calculated to create a spirituality of science score. Factor analysis on the spirituality of science scale using principal component analysis yielded a solution with a single factor extracted, with initial eigenvalues explaining 62.04% variance. Scale statistics are reported in Table 1.

We conducted a second-factor analysis to include items from all three science scales (SoS; BIS; Interest in Science), to confirm that each of the separate science scales measured distinct constructs. Principal component analysis with Varimax rotation yielded a solution with three factors extracted, with eigenvalues explaining 67.04% variance. The three extracted factors aligned with the three science scales (SoS, BIS, Interest). See Table 2 for full factor analyses. In both analyses, items from the spirituality of science scale emerged as a single factor and were distinct from other science measures.

**Table 2.** Principal Component Analysis (Varimax Rotation) of all Science Attitude Items, Study 1.

Item	Factor 1	Factor 2	Factor 3
(SoS 1) Science makes me step outside myself to a larger sense of fulfillment	**.720**	.333	.256
(SoS 2) I have had a “peak” experience while engaged in science.	**.723**	.071	.256
(SoS 3) Thinking about science brings me deep joy.	**.710**	.254	.457
(SoS 4) Science helps to show greater meaning in life.	**.707**	.410	.162
(SoS 5) There is an order to science that transcends human thinking	**.726**	.259	.102
(SoS 6) I have lost track of time while engaged in science	**.693**	.164	.351
(SoS 7) Science makes me feel deeply connected to everything.	**.792**	.308	.225
(SoS 8) Science reveals the beauty of the world we live in.	**.575**	.382	.274
(SoS 9) All things are connected through science	**.625**	.483	.129
(SoS 10) Science is a source of spirituality	**.757**	−.004	−.001
(BIS 1) Science provides us with the best understanding of the universe	.234	**.787**	.233
(BIS 2) We should only rationally believe in what is scientifically provable	.102	**.794**	.116
(BIS 3) All the tasks human beings face could be resolved by science	.228	**.748**	.001
(BIS 4) The scientific method is the most reliable way of knowing	.216	**.847**	.188
(BIS 5) Science is the most efficient means of attaining truth	.252	**.835**	.189
(Interest 1) I enjoy reading about science.	.287	.263	**.785**
(Interest 2) It is important to me to spend time thinking about scientific topics.	.356	.270	**.797**
(Interest 3) I have a strong desire to be part of the scientific community.	.448	.258	**.649**
(Interest 4) Although science is important, many other things are more important in life.	−.142	−.258	**.364**
(Interest 5) I often talk to my friends and family about scientific topics.	.304	.179	**.756**

*Note.* Strongest factor loading is indicated in bold. SoS = Spirituality of Science; BIS = Belief in Science.

#### Science Measures

Bivariate correlations were conducted between means of all measures, see Table 3. For brevity we only discuss the most relevant associations in the text. All three science measures were strongly intercorrelated (*r*s > .52, *p*s <.001), indicating convergence for general positive science attitudes.

**Table 3:** Bivariate Correlations, Study 1.

Measure	1	2	3	4	5	6	7
1. Spirituality of Science (SoS)	—						
2. Belief in Science (BIS)	.58‡	—					
3. Interest Science	.68‡	.52‡	—				
4. Need for Closure	−.10*	.01	−.16‡	—			
5. Intellectual Humility	.38‡	.32‡	.39‡	−.24‡	—		
6. Religiosity	−.23‡	−.49‡	−.24‡	.12†	−.19‡	—	
7. Spirituality	.11*	−.31‡	.02	−.04	.19‡	.56‡	—
8. Awe	.33‡	.00	.23‡	−.12†	.38‡	.21‡	.60‡

*Note. N* = 500. *p < .05. †*p* < .01. ^‡^*p* < .001.

#### Religion and Spirituality

General religiosity was negatively associated with all science attitudes (*r*s >|−.23|). Spiritual Transcendence (i.e., general spirituality) was negatively correlated with BIS (*r* = −.31, *p* < .01), but was positively correlated with the spirituality of science scale (*r* = .11, *p* < .05), and was not related to Interest in Science. This illustrates an important divergence between spirituality of science and BIS, and furthermore demonstrates spirituality of science as distinct from both other science attitudes and spirituality.

#### Awe

We predicted that feelings of awe would be related to SoS, as awe is a key emotion involved in spiritual experiences. Dispositional Awe was positively associated with both spirituality of science (*r* = .33, *p* < .001) and Interest in Science (*r* = .23, *p* < .001) but not significantly related to BIS. We followed this with a linear regression to predict Dispositional Awe from all science measures entered together. In the model, Awe was positively predicted by spirituality of science (*b* = 0.40 [0.29, 0.50], *t* = 7.60, *p* <.001) but not Interest in Science (*b* = 0.08 [−0.04, 0.19], *t* = 1.32, *p* = .19), and was negatively predicted by BIS (*b* = −0.26 [−0.324 −0.17], *t* = −5.91, *p* < .001). This suggests Awe is most closely linked to the spiritual experiences of science.

#### IH and NCC

We included IH and NCC as we were interested in cognitive openness as a general scientific value, and to compare spirituality of science with other science attitudes in a general cognitive openness ideas. NCC reflects less cognitive openness, and showed a small negative association with both SOS and BIS. IH reflects more openness to ideas, and was positively related to all science attitudes, see Table 3.

In multiple regression with all science attitudes (SoS, BIS, Interest in Science) as predictors for NCC, BIS predicted higher NCC: (*b* = 0.10 [0.02, 0.18], *t* = 2.41, *p* = .016), Interest in Science predicted lower NCC (*b* = −0.17 [−0.28, 0.07], *t* = 3.34, *p* =.001), and spirituality of science was not a significant predictor (*b* = −0.03 [−0.13, 0.07], *t* < 1). In a multiple regression to predict IH, all science attitudes independently predicted IH: spirituality of science (*b* = 0.14 [0.04, 0.23], *t* = 2.87, *p* =.004), Interest in Science (*b* = 0.20 [0.10, 0.30], *t* = 3.82, *p* < .001), BIS (*b* = 0.08 [0.05, 0.16], *t* = 2.08, *p* = .04). Overall, results suggest that cognitive openness is related to general science attitudes, but is not particular to SoS.

### Summary

Study 1 introduced the construct of spirituality of science scale. The 10-item spirituality of science scale showed strong internal reliability and items held together as a single factor in factor analyses. As expected, the spirituality of science scale was strongly correlated with other science attitudes including Interest in Science and Belief in Science. But unlike other science attitudes, the spirituality of science scale showed positive relationships with feelings of awe and spirituality. This divergence suggests that spirituality of science reflects a unique attitude toward science that is not captured by belief or interest in science, but which is characterized by its unique associations with awe and spirituality.

## Study 2: Well-Being and Meaning in the Nonreligious

If science can serve as a source of spirituality, an interesting implication is that it may provide a source of meaning and general well-being outside of religious spirituality. It is commonly reported that religiosity is associated with better psychological well-being (e.g., Diener et al., 2011; Park, 2005; Ritter et al., 2014). And a common reason suggested for this is that religion provides a source of existential support not available to nonreligious people (Adamczyk et al., 2022). But religion is not the only means to meaning, and nonreligious people can experience meaning from nonreligious sources (Galen, 2018; Speed et al., 2018; van Mulukom et al., 2023), including meaning from science (Uzarevic & Coleman, 2021). Study 2 aimed to show that differences in spirituality of science predict well-being and meaning among nonreligious people. In a survey sample of agnostic and atheist respondents, SoS was used to predict several measures of well-being: Subjective Happiness (Lyubomirsky & Lepper, 1999), Satisfaction with Life (Diener et al., 1985), Meaning in Life (Steger et al., 2006), and Stress (S. Cohen et al., 1983), and compared with Belief in Science (Farias et al., 2013) as a way of knowing. It was expected that (unlike BIS) SoS would predict stronger meaning in life and psychological well-being.

### Method

#### Participants

In all, 526 participants (232 women, 294 men, *M*_age_ = 34.7 years) were recruited from Amazon’s Turk Prime service for a small payment. We preselected participants who identified as atheist or agnostic prior to the study, and those who identified as religious diverted to an unrelated study (on religious belief and environmental attitudes).

#### Materials and Procedure

All measures were presented on 7-point Likert-type scales as described below and presented in randomized blocks.

Participants completed the five-item BIS (α = .85) and 10-item spirituality of science scale (α = .91), as in Study 1.

The *Meaning in Life Questionnaire* (MLQ) is a 10-item measure asking individuals to reflect about what makes life feel important and significant to them on a 7-point Likert-type scale, endpoints 1 = “*Absolutely Untrue*,” 7 = “*Absolutely True*” (Steger et al., 2006). The scale is comprised of two subscales: Search for Meaning in life (SM; e.g., “I am looking for something that makes my life meaningful.” α = .97) and Presence of Meaning in life (PM; e.g., “I understand my life’s meaning,” α = .96).

*Perceived Stress Scale* short form (α = .91) is a 10-item measure of perceived stress (e.g., “In the last month, how often have you felt nervous and ‘stressed’?” S. Cohen et al., 1983). Participants respond to statements on a 5-point Likert-type scale: *Never* (0), *Almost Never* (1), *Sometimes* (2), *Fairly Often* (3), and *Very Often* (4).

*Satisfaction with Life Scale* (SWL; α = .94) is a five-item measure of one’s overall life satisfaction (e.g., I am satisfied with my life), answered on a 7-point Likert-type scale, with endpoints: 1 = “*Strongly disagree*,” 7 = “*Strongly agree*” (Diener et al., 1985).

*Subjective Happiness Scale* (SHS; α = .94) is a four-item measure of self-reported happiness (Lyubomirsky & Lepper, 1999), answered on a 7-point Likert-type scale (e.g., Compared to most of my peers, I consider myself: [1] *less happy* to *more happy* [7]).

Finally, participants reported agreement on 1 to 7 Likert-type scales for three belief items: “*I believe in God*”; “*I consider myself a religious person*,” and “*I consider myself a spiritual person*.”

### Results and Discussion

All scale statistics and bivariate correlations are reported in Table 4.

**Table 4.** Bivariate Correlations, Study 2.

Measure	1	2	3	4	5	6
1. Spirituality of Science (SoS)	—					
2. Belief in Science	.40‡	—				
3. Presence Meaning	.29‡	.11*	—			
4. Search for Meaning	.19†	.002	−.31‡	—		
5. Stress scale	−.07	−.13†	−.49‡	.31‡	—	
6. Life Satisfaction	.26†	.06	.65‡	−.23‡	−.61‡	—
7. Subjective Happiness	.31†	.07	.60‡	−.16‡	−.55‡	−.69‡

*Note*.*p < .05. †*p* < .01. ‡*p* < .001.

#### Science and Religion Measures

As in Study 1, the spirituality of science scale was strongly correlated with BIS, see Table 4. We purposely selected for nonreligious participants, and means for self-reported belief in God (*M* = 1.55, *SD* = 1.13), religiousness (*M* = 1.17, *SD* = .58), and general spirituality (*M* = 2.03, *SD* = 1.61) were all near floor. Given the restricted variance in these beliefs, their correlations with other variables should be interpreted with caution. We note that BIS was negatively correlated with all three measures of belief in God (*r* = −.31, *p* < .001), religiosity (*r* = −.24, *p* < .001), and general spirituality (*r* = −.20, *p* < .001), whereas spirituality of science was positively correlated with general spirituality (*r* = .12, *p* = .007), but had no significant relation to either belief in God or religiosity.

#### SoS and Well-Being

As expected, BIS and SoS differed in their associations on the well-being and meaning measures. Spirituality of Science was positively related to almost all well-being measures, including SHS (*r* = .31, *p* < .001), SWL (*r* = .26, *p* < .001), SM (*r* = .19, *p* < .001), and PM (*r* = .29, *p* < .001), see Table 4. Notably, spirituality of science was associated with both Search for Meaning and Presence of Meaning, although these variables were negatively related to each other (if one already feels the presence of meaning, the search for meaning is less important). But for those higher in SoS, both facets of meaning are high, suggesting that the search for meaning persists despite already having experiences of meaning. Belief in Science, in contrast, was unrelated to most well-being measures, and only showed relationships with PM (*r* = .11, *p* =.011), and Stress (*r* = −.13, *p* =.003).

Because both SoS and BIS predicted variance in PM, multiple linear regression was used to predict PM with both SoS and BIS entered together. Sos remained significant in the regression model but BIS was not, see Table 5.

**Table 5:** Predicting Presence of Meaning (PM) From Spirituality of Science and Belief in Science, Study 2.

Predictors	*b*	95% CI	*t*	*p*	*sr* ^2^
Spirituality of Science	.401	[.28; .52]	6.420	<.001	.269
Belief in Science	−.014	[−.18; .15]	−.167	.867	−.007
Model: *R*^2^*=* .08, *F* (2, 523) *= 2*4.12, *p* < .001					

*Note.* Dependent variable = Presence of Meaning; CI = confidence interval.

### Summary

In a survey of atheists and agnostics, greater spirituality of science was associated with meaning and well-being measures, including Subjective Happiness, Life Satisfaction, Presence of Meaning, and Search for Meaning. This illustrates an important parallel with religious spirituality in fostering a sense of meaning in life, and benefits of SoS to nonreligious people who do not experience meaning from religious sources. Study 2 showed further evidence for a divergence between SoS and BIS as measures, as the latter generally did not predict well-being measures. But more importantly, results illustrate the important psychological benefits of using science as a source of spirituality, beyond just belief in science.

## Study 3: Engagement and Performance in Science

Another potential benefit of spirituality of science is that it promote better engagement and learning of scientific information. We tested this idea in Study 3, where participants read information on either a scientific topic (research on black holes) or non-science-related topic (applying for a mortgage) and were later tested on that information. We predicted that individuals high spirituality of science would show stronger engagement and better recall of scientific information.

### Method

#### Participants

In all, 171 undergraduate students volunteered to participate in exchange for partial credit in a psychology course (145 women, 21 men, 4 other, 1 nondisclosing; *M*_age_ = 18.8 years).

#### Measures and Procedure

Participants completed an online survey, and all items were scored on 5-point Likert-type scales. First, participants completed measures of BIS (α = .76) and spirituality of science (α = .84). The eleven-item SoS scale included the ten items as in Studies 1 and 2, adding one reverse-scored item: “I don’t feel much meaning in science.” Participants were randomly assigned to read information about either black holes (Science condition) or mortgage applications (Control condition). Information in both conditions was described on three pages, each accompanied by a related image. Participants rated how they felt during the reading on 12 different emotions (*bored, engaged, annoyed, excited, in awe, anxious, interested, confused, upset, happy, scared*). Participants then completed a five-item *Small Self scale* (α = .66; Shiota et al., 2007; e.g., “I feel the existence of things greater than myself”). Feelings of small self—where one feels tiny in comparison to something or someone greater than oneself—have been shown to be central to feelings of awe (Bai et al., 2017; Piff et al., 2015) and spiritual experiences (Preston & Shin, 2017), and so was predicted to correlate with SOS and performance on science questions. Participants next completed a four-item alternative version of the *Cognitive Reflection Task* (CRT; Thomson & Oppenheimer, 2016) to control for effects of intuitive versus reflective thinking on learning. Like the original CRT (Frederick, 2005), the alternative CRT is designed to capture a more reflective, rather than intuitive thinking style (e.g., “If you’re running a race and you pass the person in second place, what place are you in?”). We included the CRT to rule out the possibility that any observed effect of spirituality of science on science learning is explained through general scientific thinking or ability. All participants were then tested for recall of science and mortgage information, with three questions about black holes, and three questions about mortgages, based on information given in the readings. Finally, participants completed demographic information and were debriefed.

### Results

#### Emotion Factors

Factor analysis was conducted on the twelve different emotional responses to the reading, to reveal common emotional themes. Principal component analysis with Varimax rotation was used to extract factors with eigenvalues > 1. A two-factor solution emerged accounting for 56% of the variance. Factor 1 (Engagement) included loadings >.|50| on seven items: *engaged, in awe, excited, interested, happy, amused*, and *bored* (negative load). Mean ratings of these responses (reverse-score for *bored*) were calculated into a single variable, dubbed “Engagement” (α = .87). Factor 2 included positive loadings >.50 on five items: *annoyed, upset, scared, confused*, and *anxious.* Mean ratings of these responses were calculated into a single variable, dubbed “Anxiety” (α = .71).

#### Correlations

Bivariate correlations and scale statistics for all measures are reported in Table 6. As in Studies 1 and 2, means for the spirituality of science and BIS scales were strongly positively correlated (*r* = .40, *p* <.001). The Engagement emotional factor was positively correlated with both SoS (*r* = .36, *p* <.001) and BIS (*r* = .16, *p* =.03). The Anxiety factor was also correlated to SoS (*r* = .17, *p* =.03), but was not related to any other variables. The Small Self scale (Shiota et al., 2007) was used to assess feelings of personal smallness, and was positively correlated with SoS (*r* = .16, *p* =.037) and Engagement (*r* = .18, *p* =.022), but not BIS. The alternate CRT (Thomson & Oppenheimer, 2016) comprises four items (*M* = 2.64; *SD* = .97). This measure was included to differentiate spirituality of science from general intelligence or thinking styles that would facilitate learning. The CRT had a positive relationship with BIS (*r* = .20, *p* =.01) but was not significantly related to SoS, which indicates that the SoS scale is not a measure of general thinking or intellectual ability.

**Table 6.** Bivariate Correlations, Study 3.

Measure	1	2	3	4	5	6	7
1. Spirituality of Science (SoS)	—						
2. Belief in Science (BIS)	.40‡	—					
3. Small Self	.16*	.00	—				
4. Engagement factor	.36‡	.16*	.18*	—			
5. Anxiety factor	.17*	.12	−.01	.01	—		
6. CRT	.14	.20†	−.01	.06	.06	—	
7. Black Hole Correct	.18*	.09	.19*	.53‡	−.08	−.01	—
8. Mortgage Correct	.08	.06	−.10	.02	−.07	.10	−.27‡

*Note.* CRT = Cognitive Reflection Task. *p < .05. †*p* < .01. ‡*p* < .001.

#### Learning and Correct Responses

Mean correct responses to the three science (black hole) questions and three mortgage questions were calculated. As expected, more correct responses to black hole questions were given in the Science (*M* = 2.47; *SD* =.83) versus Control condition, *M* = 1.04; *SD* =.80; *F* (1, 170) = 132.01, *p* <.001; η^2^ =.44. Overall, correct responses to the black hole questions positively correlated with spirituality of science (*r* = .18, *p* =.021), mean Engagement during reading (*r* = .53, *p* <.001) and feelings Small Self (*r* = .19, *p* =.016), but not with BIS (*r* = .09, *p* =.25). As expected, more mortgage questions were answered correctly in the Control condition (*M* = 2.40; *SD* =.69) versus Science condition, *M* = 1.82; *SD* =.77; *F* (1, 170) = 26.99, *p* < .001; η^2^ =.14. Correct answers to mortgage questions were not significantly related to SoS nor any other predictors in the study, see Table 6.

#### Effects of Engagement

SoS, Engagement, and Small Self were each found to be correlated with correct science answers. As a follow up, relative contributions of these variables in predicting correct science answers were examined using linear multiple regression. With variables entered together in the model, Engagement remained a significant predictor but SoS and Small Self were not, see Table 7. This is consistent with the idea that SoS may promote science learning through stronger engagement with the material. We tested whether Engagement mediated the effect of spirituality of science on correct science answers using regression with Sobel test (Sobel, 1982). In Model 1, spirituality of science alone was shown to predict mean Engagement: *b* = 0.51; *SE* = .10, *t* = 4.98, *p* < .001, *sr*^2^=.36. In Model 2, spirituality of science and Engagement were entered together to predict correct black hole answers. Only Engagement remained significant in the regression (*b* = 0.66, *SE* = .09, *t* = 7.53, *p* < .001, *sr*^2^=.49), but SoS did not (*b* = -0.02, *t* = –0.18, *p* = .86). This indicates the effects of SoS on correct science answers could be explained via greater feelings of engagement, and the Sobel test (Soper, 2023) supported mediation through Engagement (*z* = 4.14, *p* < .001).

**Table 7:** SoS, Small Self, and Mean Engagement as Predictors for Number of Correct Science Answers.

Predictors	*b*	95% CI	*t*	*p*	*sr* ^2^
Spirituality of Science	−.042	[−.29, .20]	−.33	.74	−.022
Small Self	.158	[−.06, .37]	−1.47	.14	.096
Engagement	.643	[.47, .82]	7.30	<.001	.478
Model: *R*^2^*=* .29, *F* (3, 167) *=* 22.16, *p* < .001					

*Note*. Dependent variable = correct answers to black hole questions. CI = confidence interval.

### Summary

Study 3 examined the influence of spirituality of science on engagement and learning in science. Results provided more convergent and divergent evidence for spirituality of science: SoS was correlated with feelings of Small Self, engagement during reading, and BIS, but was not related to analytical thinking. Overall, SoS did not predict participants’ correct responses to the nonscientific information but did predict correct responses to the science (black hole) information, and this was best explained through stronger feelings of engagement.

## General Discussion

Science helps provide a deep sense of wonder, understanding, and connection that we argue here can serve as a source of spirituality for some people. Three studies investigated differences in spirituality of science and their relationship to feelings of awe, well-being, meaning, engagement and performance in science. In Study 1, spirituality of science was related to other science attitudes including Belief in Science (BIS), but only SoS predicted general spirituality and feelings of awe. In Study 2, SoS (but not BIS) predicted well-being and meaning in a group of atheists and agnostics, suggesting that scientific spirituality can provide similar psychological benefits as religious spirituality. In Study 3 people high in SoS were more engaged when reading science information, and overall SoS (but not BIS) predicted later performance on science test items. Together, these studies indicate that science can indeed be a genuine source of spirituality, with intellectual and emotional benefits.

### Future of Spirituality of Science

The intent of the present research was to establish spirituality of science as an important construct, not just as a measure, but as a way to capture the meaning experienced through science. We believe these studies do well to establish spirituality of science as a phenomenon and to show implications for well-being and learning, and also to distinguish it from other related kinds of scientific thinking and attitudes. But we consider these to be just an introduction to the topic that provides a jumping-off point for new lines of research. Studies here on science outcomes can be extended with longitudinal designs to examine retention and learning over longer periods. Here we had compared SoS with belief in science, to differentiate the spiritual aspect of science attitudes from general acceptance of science. But future research could compare spirituality of science with other related science attitudes, such as enjoyment in science or understanding of science. Studies on science and well-being may also be extended with longitudinal and experience sampling methods that could explore the downstream effects of spirituality of science on daily life satisfaction and meaning in life. Well-being research may also extend to physical health as a parallel to the benefits of religious spirituality on health (Park, 2007), consistent with past research that meaning in life is a central contributor to physical health (e.g., Nygren et al., 2005; Ryff & Singer, 1998; Wong & Fry, 1998). Future research could also compare different kinds of scientific theories and examine what aspects lend themselves to spirituality of science, especially in regard to feelings of transcendence, connection, and meaning.

Research could further examine the implications of spirituality of science as it relates to general spirituality. General spirituality is associated with moral concerns and prosociality, for example, feelings of compassion and values of universality (Einolf, 2013). Prosociality is not an inherent characteristic of spirituality but can increase in spiritual people through greater awareness and connection to others (Piff et al., 2015), and awe (Jiang & Sedikides, 2022). Could differences in spirituality of science also predict moral concerns, would spiritual experiences with science similarly activate prosociality?

Cross-cultural studies could explore the generalizability of spirituality through science where experiences and definitions of science differ culturally. Science is culturally universal; all human groups use observation and data to make inferences about the natural world and to solve the practical problems that affect their lives. In larger industrialized cultures, concepts of science may be strongly linked with technology and cutting-edge advances, but in agrarian and tribal cultures concepts of science may be more directly tied to nature as it applies to the practical concerns in daily life. So too might ideas of spirituality of science in these cultures, and cross-cultural research can help to establish different meanings of spirituality of science, and where it might meld into other kinds of spirituality. Western cultures may also be more likely to actively separate practices of science and religion, where in other cultures they are seen as entirely compatible as practices that enrich rather than contradict each other (McPhetres et al., 2021; Rios & Aveyard, 2019). In this case, spirituality of science may be common in other cultures as well and could be associated with higher religiosity where science and religion are seen as compatible.

### Spirituality by Any Other Name

Our argument that science serves as a source of spirituality may seem a radical or contradictory approach. How can spirituality be derived without reference to belief in God, the supernatural, or some other aspect of the scared? But we argue (as others have) that spirituality can be independent of any supernatural belief. Rather, we specify spirituality as marked by feelings of meaning, connection, and profound transcendent emotion. These elements of spirituality can apply to the supernatural and be derived from personal belief in God, but can also apply to experiences with science and be without any supernatural element.

But we consider *sacredness* perception as an integral part of spirituality in general, and of spirituality of science. Sacredness itself has been notoriously tricky to define; however, the best definitions of sacredness characterize it as some quality of “specialness” to be set apart from ordinary things (Pargament et al., 2017), and characterized by a set of sacred qualities: transcendence, ultimacy (perception of truth), and boundlessness (beyond space and time; see also Pargament & Mahoney, 2005). These elements overlap with our own operationalization of spirituality in its application to science and are reflected in the items on the spirituality of science scale. Importantly, perceptions of sacredness apply to both a core of the divine (e.g., God) and to ordinary things which have taken on qualities of the sacred (Pargament et al., 2017). Sacredness can therefore be extended to science as well as other domains (e.g., art, personal relationships) providing those experiences are characterized by similar transcendent emotions and sense of connection and purpose. For example, experiences in nature are often cited as a source of spirituality (Preston & Shin, 2017) and the sacred (Delaney, 2005), as it can evoke these feelings of transcendence and connection to the universe. That said, science possesses some unique attributes that particularly lend itself to experiences of spirituality. Science and religion share a common explanatory function for that lends itself to creating meaning (Preston & Epley, 2005). Other kinds of experiences (like art) might also have transcendent moments of awe or gratitude but may not fulfill existential functions. This does not mean spirituality cannot be found in other domains, only that science may be particularly adept in eliciting the sense of coherence underlying spirituality.

### Spirituality and Science and Religion

The idea of spirituality of science should be distinguished from other issues surrounding the relationship between science and religion. Psychology research on the relationship between science and religion has often focused on their roles as different kinds of explanatory systems for understanding the world (Davoodi & Lombrozo, 2022), and the extent that these systems are viewed as competing or complementary (e.g., Legare et al., 2012; Preston & Epley, 2009; for a discussion, see Rutjens & Preston, 2020). Here we offer a different approach: that like religion, science can be a source of spirituality. Indeed the connection between science and spirituality may be closer than between science and religion. The science–religion relationship tends to focus on external factors such as their roles as explicit belief systems. But science can be directly linked to spirituality through *internal* experiences of meaning and awe. Indeed, if there is any meaningful point of connection between science and religion, it may be through a shared sense of spirituality that they can each evoke.

Further research should also explore the relationship between scientific spirituality and religious spirituality. In the present studies, we observe a negative correlation between SoS and general religiosity. But this does not mean the two are incompatible forms of spirituality—it is certainly possible to experience spirituality through both science and religion. How might a person deal with these two sources of spirituality? Religious people can already derive meaning from their religious beliefs, but perhaps using science as an additional source of spirituality could further boost the meaning and well-being in a religious person. But it is alternatively possible that this would be redundant with the meaning provided by religion. However, we suspect that science can be seen as a way of enhancing religious spirituality. Indeed, religious people see less conflict between religion and science (Leicht et al., 2021) one way that science and religion may be seen as compatible is through their common spirituality.

## Conclusion

Although science and religion differ in many ways, they share a capacity for spirituality through feelings of awe, coherence, and meaning in life. This capacity for spirituality has some important benefits and implications, as we find here. People with greater feelings of Spirituality of Science were more positively engaged with science material, which predicted better science performance. And in a group of atheists and agnostics, Spirituality of Science predicted measures of well-being and meaning in life, paralleling the positive effects of religion that is frequently observed in religious people. This work contributes not only to our current understanding of science attitudes but also to our general understanding of spirituality.
